# Reactivation-dependent transfer of fear memory between contexts requires M1 muscarinic receptor stimulation in dorsal hippocampus of male rats

**DOI:** 10.1101/lm.054039.124

**Published:** 2024-09

**Authors:** Karim H. Abouelnaga, Andrew E. Huff, Kristen H. Jardine, Olivia S. O'Neill, Boyer D. Winters

**Affiliations:** Department of Psychology and Collaborative Neuroscience Program, University of Guelph, N1G 2W1, Guelph ON, Canada

## Abstract

Memory updating is essential for integrating new information into existing representations. However, this process could become maladaptive in conditions like post-traumatic stress disorder (PTSD), when fear memories generalize to neutral contexts. Previously, we have shown that contextual fear memory malleability in rats requires activation of M1 muscarinic acetylcholine receptors in the dorsal hippocampus. Here, we investigated the involvement of this mechanism in the transfer of contextual fear memories to other contexts using a novel fear memory updating paradigm. Following brief reexposure to a previously fear conditioned context, male rats (*n* = 8–10/group) were placed into a neutral context to evaluate the transfer of fear memory. We also infused the selective M1 receptor antagonist pirenzepine into the dorsal hippocampus before memory reactivation to try to block this effect. Results support the hypothesis that fear memory can be updated with novel contextual information, but only if rats are reexposed to the originally trained context relatively recently before the neutral context; evidence for transfer was not seen if the fear memory reactivation was omitted or if it occurred 6 h before neutral context exposure. The transferred fear persisted for 4 weeks, and the effect was blocked by M1 antagonism. These findings strongly suggest that fear transfer requires reactivation and destabilization of the original fear memory. The novel preclinical model introduced here, and its implication of muscarinic receptors in this process, could therefore inform therapeutic strategies for PTSD and similar conditions.

Memory updating is a fundamental process that facilitates the integration of novel information into preexisting memory traces, thereby maintaining their relevance and accuracy over time (Hupbach et al. 2007; Lee 2009; McKenzie and Eichenbaum 2011). This is crucial for adaptive behaviors, enabling individuals to adjust to changing environmental demands. However, this process could be exploited in the case of certain maladaptive memories, especially those rooted in fear or trauma. For example, a characteristic feature of contextual fear memories is generalization, whereby fear responses extend beyond the original fear-inducing stimulus to a broad spectrum of related stimuli, exacerbating the distress and dysfunction experienced by affected individuals (Dunsmoor et al. 2009; Lissek et al. 2014). The basis for such generalization remains unclear. However, it is possible that memory-updating mechanisms could lead to the linking of aversive memories to neutral contexts, contributing to some forms of generalization. It is therefore important to consider the environmental and neurobiological factors that could drive such associations.

Consolidated memories can become labile upon reactivation (Nader et al. 2000; Jardine et al. 2022), and this renewed malleability is related to underlying mechanisms of synaptic destabilization (Lee et al. 2008; Wideman et al. 2018). We have recently implicated the cholinergic system in reactivation-induced destabilization of object, spatial, and contextual fear memories in rats; specifically, M1 muscarinic acetylcholine receptor (mAChR) activity appears to be necessary for destabilization of these memory types (Stiver et al. 2015, 2017; Huff et al. 2022; Abouelnaga et al. 2023; Wideman et al. 2023). Furthermore, direct integrative updating of object memories with new contextual information was similarly blocked when M1 mAChRs were antagonized before reactivation of the original object memory (Jardine et al. 2020). These findings suggest that mAChRs are involved in the process of memory updating because of their permissive role in memory destabilization (Jardine et al. 2020). The goal of the present study was to demonstrate a similar behavioral process—and mechanistic basis—for the updating of contextual fear memories with novel, previously neutral, contextual information.

To this end, here we introduce a novel paradigm, the fear reactivation and transfer (FRaT) task, which models fear memory updating in rats through the process of memory linking following the reactivation of a contextual fear memory. While there is evidence that the introduction of certain types of information, aversive or positive, can update fear memories (Haubrich et al. 2015; Grella et al. 2022, Zaki et al. 2023), to our knowledge, there is no task that models fear memory linking to other contexts, which is a hallmark characteristic of certain maladaptive memories. We hypothesized that the introduction of neutral contextual information immediately following fear memory reactivation leads to the integration of such contextual information with the previously consolidated memory, resulting in fear transfer. Moreover, given our previous report implicating dorsal hippocampal M1 mAChRs in contextual fear memory destabilization (Abouelnaga et al. 2023), we predicted that antagonizing these receptors should block the updating effect by preventing destabilization of the original contextual fear memory.

## Results

### Histology

**Figure 1.**
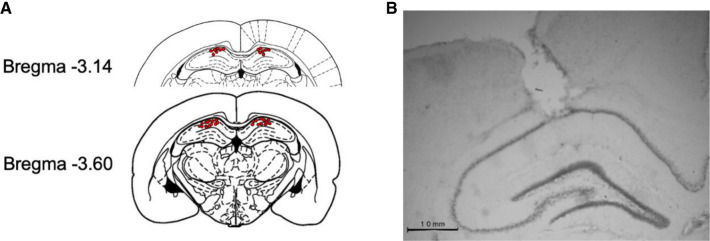
Schematic representation of the cannula placements for intra-dHPC infusions. (*A*) Individual placements for each subject included in Experiment 5 (*n* = 21). (*B*) Micrograph of an example cannula tip placement terminating in the CA1 region of the dHPC.

All rats that participated in behavioral testing in Experiment 5 were implanted with a bilateral cannula in which the tips were targeting the CA1 region in the dHPC. To verify placements, the rats were anesthetized with 0.8 mL of Euthansol (82 mg/mL, Kirkland) and then underwent pericardial perfusions with PBS and 4% formalin. Brains were then extracted and placed in a formalin solution for a minimum period of 24 h before being transferred to a 20% sucrose solution for brain slicing preparation. Brains were sliced with a cryostat to 50 μm in width and then every third slice was mounted on a gelatin-coated slide. The slides were then thionin stained and visualized under the microscope (Fig. 1B). Placements were marked as individual dots, and a representative micrograph was obtained of the infusion tip in the CA1 region (Fig. 1A).

### Contextual fear transfers to a neutral context presented following reactivation of the original memory

**Figure 2.**
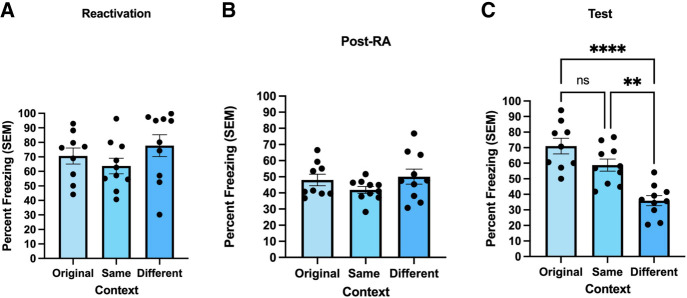
Contextual fear transfers to an alternate context when the alternate contextual information is introduced following reexposure to the training context. (*A*) During the reactivation session, freezing levels did not differ between the three groups. Freezing behavior was measured as percent freezing (±SEM) across a 90 sec reactivation session. (*B*) There were also no differences in post-reactivation (Post-RA) context freezing. Freezing behavior was measured as percent freezing (±SEM) across an 8 min post-reactivation session in the alternate contexts. (*C*) In the test session, rats exposed to the same alternate context as the post-RA session (Same) froze significantly more than those tested in the different alternate context (Different). Same alternate context rats also froze similarly to the rats that were tested in the original fear conditioning chamber (Original). Freezing behavior (*n* = 9 per group for “Original” condition and *n* = 10 per group for “Same” and “Different” conditions) was measured as percent freezing (±SEM) across an 8 min test session. (****) *P* < 0.0001, (**) *P* < 0.01.

The FRaT task was used in this experiment to determine whether contextual fear memories incorporate new contextual information if such information is presented immediately following reactivation. On reactivation day, results of a one-way between-subjects ANOVA showed no significant difference in freezing levels between the three groups [*F*(2,26) = 1.284, *P* = 0.294, η^2^= 0.090; Fig. 2A]. Additionally, there was no significant difference in freezing behavior in the post-reactivation alternate context [*F*(2,26) = 1.460, *P* = 0.251, η^2^= 0.101; Fig. 2B].

During the test session, rats exposed to the same alternate context as in the post-reactivation session had significantly higher freezing levels compared to rats tested in a different alternate context (Fig. 2C). Results of a one-way between-subjects ANOVA indicated a significant effect of context [*F*(2,26) = 19.276, *P* < 0.001, η^2^= 0.597]. Specifically, Tukey's multiple comparisons test revealed that rats exposed to the same alternate context on test day (Same) froze significantly higher than those tested in a different alternate context (Different) (*P* = 0.001), but did not differ from rats placed back in the fear conditioning chamber where the original contextual fear memory was acquired (Original) (*P* = 0.105).

### Transfer of fear to a neutral context requires reactivation of the original fear memory

The purpose of this experiment was to determine whether reactivation of a contextual fear memory is necessary for the transfer of fear to an alternate context. Here, only the “same” condition was tested. One group underwent the reactivation session by being placed back into the fear conditioning chamber before being exposed to the alternate context (Same-RA), while the other group was placed into the alternate context without first being reexposed to the training context (Same-NoRA).

**Figure 3.**
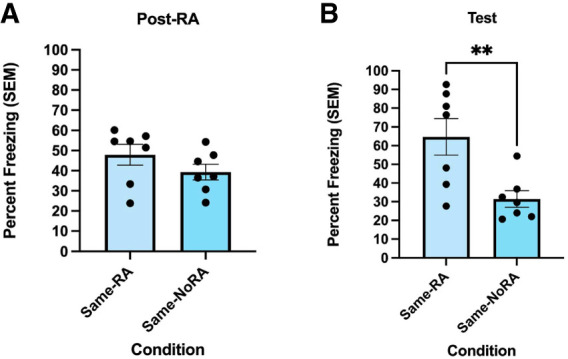
The transfer of fear to alternate contexts is reactivation-dependent. (*A*) During the reactivation (RA) session, there was no significant difference in freezing behavior between the groups in the alternate contexts. Freezing behavior was measured as percent freezing (±SEM) across an 8 min post-reactivation session in the alternate contexts. (*B*) For the test session, the group that was reexposed to the fear conditioning chamber during the reactivation session (Same-RA) froze significantly higher in the alternate context than the group that was not exposed to the chamber (Same-NoRA). Freezing behavior (*n* = 7 per group) was measured as percent freezing (±SEM) across an 8 min test session. (**) *P* < 0.01.

Results of an independent-samples *t*-test showed no significant difference in freezing behavior between groups in the alternate contexts on reactivation day [*t*(12) = 1.321, *P* = 0.211; Fig. 3A]. On test day, the group that received a reactivation session in the fear conditioning chamber froze significantly higher in the alternate context compared to the group that did not [*t*(12) = 3.102, *P* = 0.0092; Fig. 3B], suggesting that the fear transferred to an alternate context only when the original memory was reactivated.

### Reactivation-mediated fear transfer persists for at least 4 weeks

**Figure 4.**
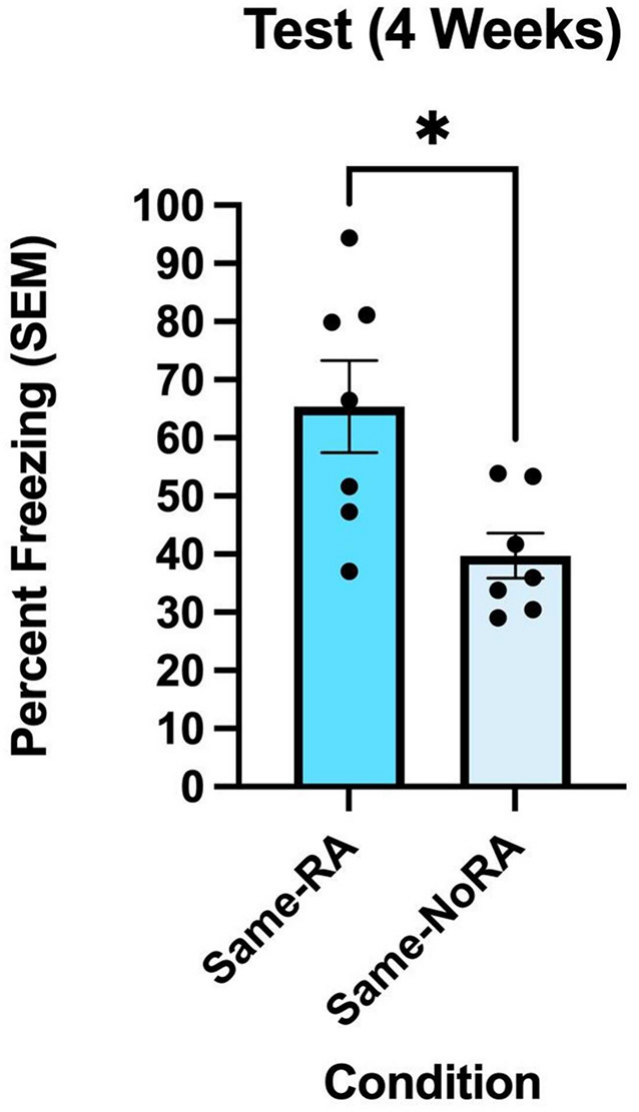
Transfer of fear to an alternate context is persistent when tested 4 weeks later. During the testing session, the Same-RA group from the previous experiment froze significantly higher than the Same-NoRA group, indicating that the transfer of fear to an alternate context is persistent. Freezing behavior (*n* = 7 per group) was measured as percent freezing (±SEM) across an 8 min test session. (*) *P* < 0.05.

To determine the persistence of the fear transfer effect in the FRaT task, the same rats underwent a retest session in the same alternate contexts that they were exposed to in the previous experiment. Results of an independent-samples *t*-test indicated a significant difference in freezing behavior between groups during the test session [*t*(12) = 2.909, *P* = 0.013; Fig. 4], suggesting that the rats still experienced the alternate context as aversive even after 4 weeks.

### Contextual fear transfer occurs within a limited time window following reactivation

**Figure 5.**
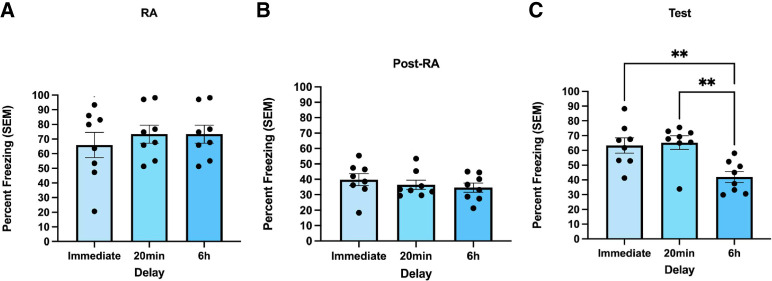
Fear transfer only occurs if exposure to the alternate context happens within the 6 h reconsolidation window. (*A*) During the reactivation session, freezing levels did not differ between the three groups. Freezing behavior was measured as percent freezing (±SEM) across a 90 sec reactivation session. (*B*) There were no significant differences in freezing behavior during the post-reactivation sessions. Freezing behavior (*n* = 8 per group) was measured as percent freezing (±SEM) across the 8 min post-reactivation session. (*C*) During the test session, the groups exposed to the alternate context immediately (immediate) or 20 min post-reactivation (20 min) froze significantly higher than the group exposed to the alternate context 6 h (6 h) post-reactivation. Freezing behavior (*n* = 8 per group) was measured as percent freezing (±SEM) across the 8 min test session. (**) *P* < 0.01.

The purpose of this experiment was to determine whether this fear transfer effect only occurs within the putative reconsolidation window. Additionally, we aimed to determine whether this effect only happens if exposure to the alternate context occurs immediately after memory reactivation. Thus, we exposed rats to alternate contexts at one of three different delays: immediately after reactivation of the contextual fear memory (Immediate), 20 min after reactivation (20 min), or 6 h after reactivation (6 h). On reactivation day, results of a one-way between-subjects ANOVA indicated no significant differences in freezing levels in the fear conditioning chamber [*F*(2,21) = 0.367, *P* = 0.697, η^2^= 0.034; Fig. 5A]. Additionally, there were no significant differences in freezing in the alternate contexts between the three groups [*F*(2,21) = 0.615, *P* = 0.550, η^2^= 0.055; Fig. 5B].

During the test session, rats exposed to the alternate context immediately or 20 min after reactivation froze significantly more than those that saw the alternate context 6 h post-reactivation. A one-way between-subjects ANOVA revealed a significant effect of delay on freezing levels [*F*(2,21) = 7.987 *P* = 0.003, η^2^= 0.432; Fig. 5C]. Specifically, a Tukey's multiple comparisons test revealed that rats exposed to the alternate context immediately post-reactivation (Immediate) froze significantly higher than those placed into the alternate context 6 h later (6 h) (*P* = 0.0091). However, there was no significant difference between the immediate group and the group seeing the alternate context 20 min later (20 min) (*P* = 0.9512).

### M1 mAChR antagonism in dHPC blocks reactivation-dependent contextual fear transfer

**Figure 6.**
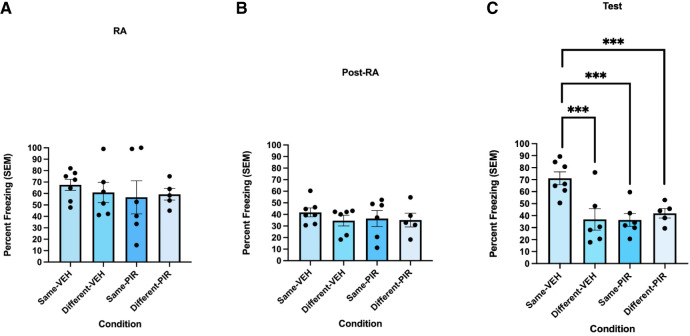
Contextual fear transfer requires M1 mAChR activity in the CA1 region of the dHPC. (*A*) During the reactivation session, freezing levels did not differ between the three groups. Freezing behavior was measured as percent freezing (±SEM) across a 90 sec reactivation session. (*B*) There was no significant difference in freezing behavior during the post-reactivation session. Freezing behavior was measured as percent freezing (±SEM) across the 8 min post-reactivation session. (*C*) The Same-PIR group froze significantly less than the Same-VEH group during the test session, implying that PIR disrupted the updating of the fear memory to incorporate alternate contextual information. Freezing behavior was measured as percent freezing (±SEM) across the 8 min test session. (***) *P* < 0.001.

Given the involvement of M1 mAChRs in the destabilization of contextual fear memories, as well as the role of the dHPC in this process (Abouelnaga et al. 2023), we predicted that the updating of contextual fear memories would also be dependent on dHPC M1 mAChR stimulation. Here, we directly antagonized these receptors in the dorsal CA1 during memory reactivation. On reactivation day, a 2 × 2 between-subjects ANOVA showed no significant interaction between drug and context [*F*(1,20) = 0.248, *P* = 0.624, η^2^ = 0.012]. Additionally, there was no significant effect of pirenzepine on reactivation day freezing levels in the fear conditioning chamber [*F*(1,20) = 0.049, *P* = 0.827, η^2^ = 0.002; Fig. 6A]. Post-reactivation freezing in the alternate context was not significantly affected when pirenzepine was infused 30 min before reactivation [*F*(1,20) = 0.617, *P* = 0.442, η^2^ = 0.030], nor was there an effect of post-reactivation context [*F*(1,20) = 0.208, *P* = 0.653, η^2^ = 0.010; Fig. 6B]. Additionally, there was no significant interaction between the drug-infused pre-reactivation and the context the rats were exposed to post-reactivation [*F*(1,20) = 0.298, *P* = 0.591, η^2^ = 0.015].

During the test session, a 2 × 2 between-subjects ANOVA revealed a significant interaction between drug infused before reactivation and the context that the rats were exposed to post-reactivation [*F*(1,20) = 9.828, *P* = 0.005, η^2^ = 0.329; Fig. 6C]. An analysis of simple main effects revealed that the drug administered significantly affected freezing where the group receiving PIR froze significantly less than the group receiving VEH [*F*(1,20) = 15.822, *P* < 0.001, η^2^ = 0.442; Fig. 6C]. Additionally, the group that saw the same context froze significantly higher when receiving VEH than when getting PIR [*F*(1,20) = 16.283, *P* < 0.001, η^2^ = 0.449; Fig. 6C]. Lastly, there was no significant difference between the groups that received PIR if they saw same or different contexts [*F*(1,20) = 0.363, *P* = 0.554, η^2^ = 0.018; Fig. 6C].

## Discussion

In this investigation, we introduce a novel approach to studying the transfer of learned fear to other contexts, with a specific emphasis on the importance of reactivation of the original memory and the role of M1 mAChRs in male rat. Our findings, derived from the novel FRaT task, suggest that additional contextual information can be incorporated into an existing fear memory and that this memory modification is reactivation- and time-dependent.

First, we used the FRaT task to update contextual fear memory in rats, apparently incorporating information from a neutral context presented shortly after reactivation of the original fear memory. This finding is consistent with reconsolidation theory, which posits that upon reactivation, memories temporarily enter a labile state, making them susceptible to modification (Nader et al. 2000). We have previously described a similar phenomenon for object memory updating in rats (Jardine et al. 2020). Updating of contextual fear memory with new information has been explored in other studies investigating the linking of aversive experiences across time (Jung et al. 2023; Zaki et al. 2023). Furthermore, others have demonstrated the possibility of updating an established contextual fear memory with appetitive information, suggesting the versatility and adaptability of memory processes (Ramirez et al. 2013; Haubrich et al. 2015). However, here we demonstrate updating of a contextual fear memory without altering the original conditioned response, as seen in Experiment 1 where rats were still freezing in the fear conditioning chamber on test day even if alternate contextual information had been introduced post-reactivation.

Behavior consistent with contextual fear transfer was only seen in the FRaT task when rats were exposed to the original conditioned context shortly before alternate context presentation; no updating occurred when the original context exposure was omitted or when the reactivation occurred 6 h before alternate context exposure. The reactivation- and time-dependence of this effect is significant as it delineates the specificity of the memory updating process. It suggests that the rats are not indiscriminately generalizing their fear to any context they encounter, but rather, the reactivation primes the original memory for subsequent modification. This interpretation is also supported by the relatively lower freezing levels in the different alternate context condition on test day. This distinction between memory updating and generalized fear is crucial for understanding the nuances of memory processes operating here. If the rats were simply generalizing their fear to any context through fear memory updating, it would imply a broad and nonspecific response. However, our results indicate a more targeted and specific process, whereby the reactivated original memory is modified to incorporate new information presented within a restricted period of lability (Nader et al. 2000). It is also worth noting that rats in the various conditions did not differ in terms of freezing either during the memory reactivation session or when they were placed into the alternate contexts following reactivation; this pattern reinforces our interpretation of the present results in terms of a specific memory reconsolidation-based process of fear transfer. Additionally, our results showing that freezing in the alternate context persists when tested 4 weeks later suggest a lasting, rather than temporary, effect. A shorter-term effect might indicate a less specific effect related to the recent exposure to the original context. This alternative explanation is also not supported by our failure to see enhanced freezing when rats were tested in the different alternate context condition.

It is important to note that the effect seen in the FRaT task could potentially be attributed to some sort of second-order conditioning. Because the alternate contexts are presented post-reactivation, it is possible that this contextual information becomes associated with the original context, or the response or fear state produced by reexposure to this context. For example, second-order conditioning can occur for auditory fear (Debiec et al. 2004, 2006; Payne et al. 2023). Specifically, Debiec et al. (2006) paired a previously conditioned tone (CS1) with a new tone (CS2) and showed that animals will freeze to CS2 after several such pairings. However, the procedures in Debiec et al. (2006) differed significantly from the present methods. Here, the rats were exposed to the putative CS2, the alternate context, FOLLOWING the exposure to CS1. Moreover, the fact that fear spreads to the alternate context when it is presented 20 min following exposure to the original context, suggests that the effect observed in the FRaT task is not the result of backward second-order conditioning, for which there is very little actual evidence, and which would likely require a much shorter delay between context presentations (Mowrer et al. 1988; Barnet et al. 1991). Additionally, the short duration of the reactivation session makes it less likely that the rats established a second-order association between the contexts following a single pairing. Nonetheless, it is possible that a lingering fear state produced by reexposure to the training context was associated with the alternate context during its presentation shortly after the reactivation session, and this could also explain the effects observed here. Testing the effects of extinction could possibly help to distinguish between these explanations, as extinguishing freezing to the alternate context might not affect freezing to the training context if second-order conditioning underlies the present results; this is an important question for future investigation.

A final vital distinction between the FRaT task and similar procedures used in Debiec et al. (2006) and Payne et al. (2023) is that in the current study, the rats were previously habituated to the alternate contexts (the putative CS2). We did this to reduce the likelihood of nonspecific fear generalization when presenting a new context shortly following the reactivation of an aversive memory. The latent inhibition literature indicates that such preexposure to a context decreases the likelihood of the context subsequently becoming associated with an aversive stimulus (Lubow and Moore 1959; Blanchard et al. 2010). Thus, previous evidence, combined with the reactivation- and time-dependence of the current effects, support the interpretation that the FRaT task reflects fear memory updating; however, future studies are needed to more confidently identify the underlying behavioral basis of these effects.

The neural mechanisms underlying the present effect remain unclear. However, linking of memories through coactivation of neuronal ensembles seems a likely explanation. Cai et al. (2016), for example, demonstrated that memories for contexts encoded closely in time (5 h) could be linked apparently via overlapping neural ensembles. Furthermore, Zaki et al. (2023) report that strong fear conditioning in one context can lead to later offline reactivation of the neural ensemble associated with the trained context as well as that for a previously presented (2 days earlier) neutral context, resulting in retrospective linking of the learned fear with that neutral context. While a similar process might be at play in the current study, the present behavioral effects are unique in that they demonstrate a critically reactivation-dependent form of prospective memory linking. Indeed, fear transfer in the FRaT task was not observed if the original contextual fear memory was not explicitly reactivated. The explicit reactivation session used here could accomplish something similar to the ensemble coactivation implicated by Zaki et al. (2023), but possibly in a more selective and prospective manner. The effect reported by Zaki et al. (2023) was only observed retrospectively and not seen when a relatively low shock protocol was used for the initial fear conditioning. Here, fear transfer occurred in a highly reactivation- and time-dependent manner. Moreover, despite both alternate contexts being preexposed 72 h before the fear conditioning session (to habituate rats to these contexts), fear only transferred to the specific alternate context presented following reexposure to the trained context. Thus, a generalized linking of fear with contexts explored within a few days of the training event did not occur; the original fear memory was only updated with the specific contextual information presented within a specific window of time following memory reactivation. There is one earlier report of apparent memory linking following reactivation, whereby fear conditioning to a second tone was enhanced when conducted shortly after presentation of a different tone previously paired with shock (Rashid et al. 2016). In contrast, however, here we provide evidence for memory reactivation- and time-dependent transfer of a fear response to an otherwise neutral context.

The fear memory updating effect reported here requires mAChR stimulation. Here, we targeted the dorsal CA1 with a selective M1 mAChR antagonist to block the destabilization of the contextual fear memory when reactivated in the original fear conditioning chamber. The fact that pirenzepine blocked reactivation-dependent transfer of fear to the alternate context is consistent with the implication of M1 mAChRs in many forms of memory destabilization (Stiver et al. 2015, 2017; Huff et al. 2022; Wideman et al. 2023; Abouelnaga et al. 2023), as well as our current hypothesis that a reconsolidation-based mechanism underlies the behavioral effect in the FRaT task. Additionally, our past work suggests that M1 mAChR activity during memory reactivation could lead to downstream activation of CaMKII and the ubiquitin-proteasome system (Stiver et al. 2017; Wideman et al. 2023), consistent with a documented role for synaptic protein degradation in memory destabilization (Lee et al. 2008; Jarome et al. 2011). The plasticity resulting from such a process, combined with the coactivation of contextual representations (ensembles) in the brain could facilitate updating of the original fear memory with the new contextual information, leading to fear transfer.

The FRaT task represents a novel animal model to facilitate the study of fear memory transfer between contexts and its behavioral and biological bases. To this end, the current study presents clear avenues for further research into the role of the cholinergic system, specifically M1 mAChRs, in fear memory updating. Elucidating such memory modification mechanisms has potential implications for better understanding and treatment of conditions like post-traumatic stress disorder (PTSD) in which maladaptive memory transfer could underlie certain debilitating symptoms. It is also important to acknowledge that only male rats have been used in this study to establish the FRaT task as a valid protocol for investigating the transfer of fear memory to alternate contexts. Future directions should investigate whether male and female rats display memory linking according to similar behavioral and neurobiological mechanisms. The present results suggest that memory linking could occur through processes involved in the reactivation and updating of the original traumatic memory and that interventions targeting this process could be effective in preventing the unfortunate spread of fear to stimuli not originally associated with the initial episode.

## Materials and Methods

### Subjects

Seventy-five male Long-Evans rats were obtained from Charles River, QC and arrived weighing between 150 g and 250 g. Testing began when rats reached an approximate weight of 275 g. Upon arrival, they were placed in standard rectangular cages (48 × 26 × 20 cm) composed of polycarbonate and containing Envirodry bedding and standard enrichment (nesting materials and wooden block). They were housed with a reversed light–dark cycle in which (lights off 8:00–20:00). Rats were acclimated to the room for 1 week before experiencing researcher handling in preparation for the experiments. Food and water were available ad libitum. Behavioral testing took place during the dark phase. Most experiments used 8–10 rats, with a minimum of six rats, outliers included. Our previous work has indicated that a power of 0.80 can be achieved using a minimum sample size of six rats (α = 0.05). All procedures were approved by the Animal Care Committee of the University of Guelph.

### Surgical procedures

For Experiment 5, each rat was implanted with cannulas in the dHPC. Twenty-two gauge indwelling guide cannulas (Plastics 1, HRS Scientific, Quebec) were implanted bilaterally in the CA1 region of the dHPC. Before surgery, rats were anesthetized using isoflurane (Benson Medical Industries, Markham, Ontario) and then subcutaneous injections of Metacam (5 mg/kg) for inflammation reduction, slow-release buprenorphine (1.2 mg/kg) for pain relief, 0.9% saline, and lidocaine (20 mg/kg) as a topical anesthetic. Following that, the head was shaved and a vertical incision (3–4 cm) was made to expose the skull. The skin was retracted and Bregma visualized using Q-tips covered with hydrogen peroxide. Following the measurement of Bregma, four screws were drilled in opposing ends of the skull and a guide cannula was dropped at the designated coordinates for area CA1 relative to Bregma (Paxinos and Waston 2007); anterior–posterior −3.8 mm, medial/lateral: ± 2.5 mm, and dorsal/ventral: −2.5 mm. The cannula was then stabilized using a combination of dental cement and jet liquid. Dummy cannulas (0.36 mm) were inserted into the guide cannulas before the rat was placed in a recovery cage under heat for a period of 30 min. Once the rat was awake and bright, alert, and responsive, he was moved to his home cage to recover for a minimum of 7 days.

### Micro-infusion procedure

Micro-infusions took place in a room separate from where behavioral testing was performed. The dummy cannulas were removed while the rat was gently restrained, and 28 gauge infusion cannulas were inserted into the guide cannula. Bilateral infusions were conducted using two Hamilton syringes connected to a Harvard Apparatus precision pump. The infusion process lasted for 2 min. Following this, the infusion cannulas were removed, and the dummy cannulas were reinserted. All animals were habituated to the infusion process for 2 days before the training procedure commencing; during these sessions, dummy cannulas were removed and the infusion cannulas inserted, but no fluid was infused.

### Drug administration: intracranial

Pirenzepine (Sigma-Aldrich), a selective M1 mAChR antagonist was given in Experiment 5. It was administered at a dose of 20 μg/μL 30 min before reactivation, which has previously been shown to block destabilization of object location memories in dHPC (Huff et al. 2022). Physiological saline (0.9%) was used as a control (VEH) in each experiment.

### Fear conditioning apparatus

Four fear conditioning boxes (30 × 24 × 24 cm), each housed within separate sound-attenuating chambers (Med Associates Inc., Fairfax, VT), were used for all experiments. Near-infrared imaging was accomplished via a camera mounted inside the door of each chamber. The camera captured movement at 30 frames per second and automatically scored freezing through Video Freeze software (Med Associates Inc.). Throughout the experiments, the house light was always on in the boxes. Between rats, boxes were cleaned with 5% hydrogen peroxide (H_2_O_2_).

### Fear reactivation and transfer task

**Figure 7.**
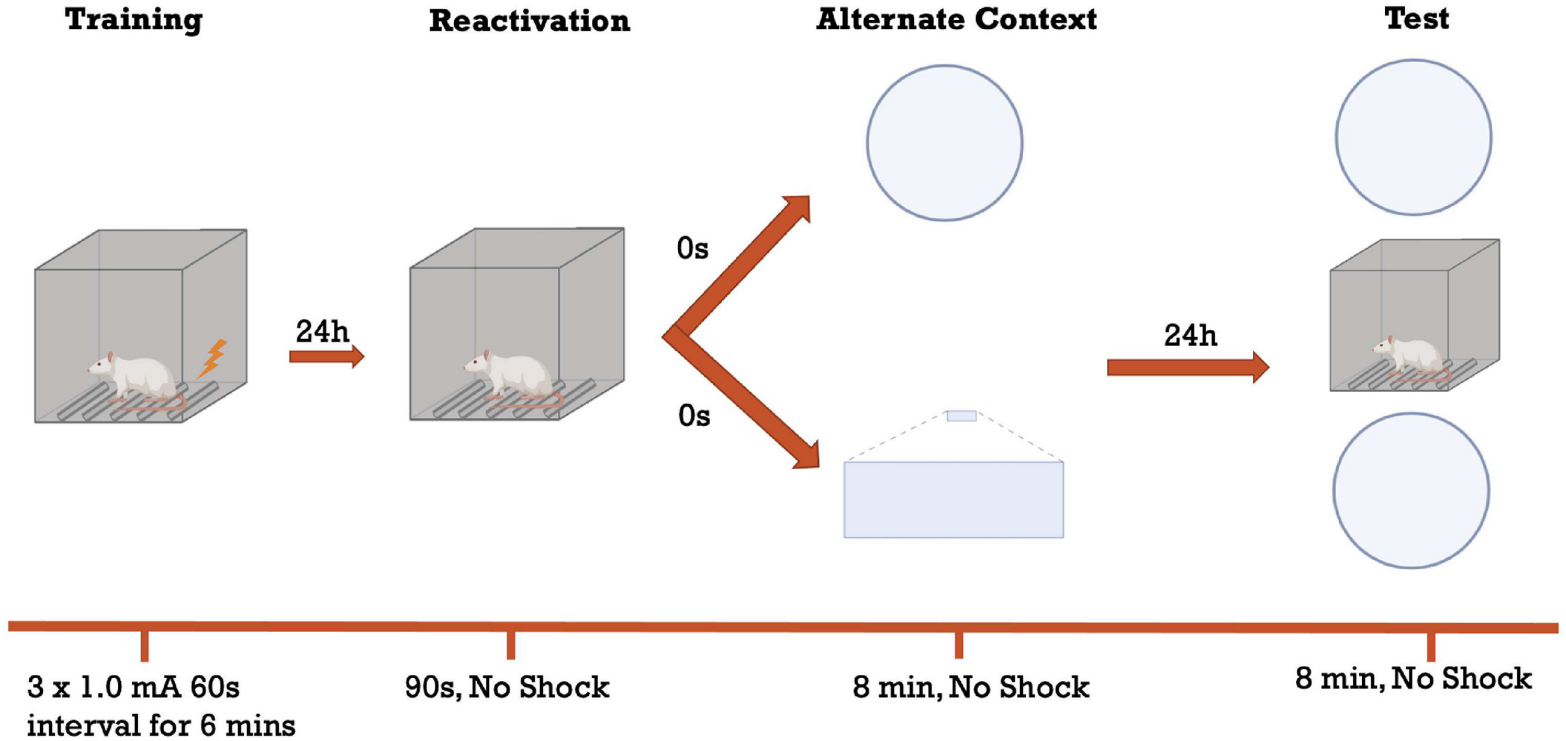
Illustration of the FRaT task. Rats undergo a training session in which they receive three footshocks. The reactivation session occurs 24 h later and lasts 90 sec in the fear conditioning chambers. Animals are exposed immediately after to either a circular or triangular alternate context for 8 min before being returned to their home cages. Twenty-four hours later, they are assessed for freezing in either the same alternate context as the day before, a different alternate context, or the original fear conditioning chamber for a period of 8 min.

The FRaT task (Fig. 7) consisted of three sessions run 24 h apart. Before these, all rats were habituated to the alternate contexts (triangle and circle apparatuses) in counterbalanced order and on separate days for a period of 8 min in each apparatus. The triangle apparatus walls were made of white corrugated plastic, with a smooth black rubber floor. The posterior wall was 75 cm long, and the other two walls were 60 cm long; all walls were 60 cm tall. The circle apparatus was made of gray plastic, with a floor made of black fine-grain waterproof sandpaper, reaching 48 cm in height with a diameter of 53 cm.

The training session took place 72 h following the second habituation day. For this session, the rats were placed in the fear conditioning chamber for a 2 min baseline period during which no footshocks occurred. Following this, three footshocks (1 mA, 1 sec each) were delivered 60 sec apart. This was followed by a 2 min post-shock period before the rat was removed from the box. The total training session time was 6 min.

Twenty-four hours later, the contextual fear memory was reactivated for each rat in its original training box by placement in the chamber for a 90 sec period. There were no footshocks delivered in this session. Immediately after, the rats were placed in one of two alternate contexts, either the circle or triangle, counterbalanced, for an 8 min period during which they were free to explore; no footshocks were delivered.

The following day, the rats were tested for freezing in one of three contexts. They were either exposed to the same alternate context as the one they saw on the reactivation day, or a different alternate context. Alternatively, some rats were placed back into the fear conditioning box to assess the original memory.

### Experimental approach

#### Habituations to alternate contexts

Before the initiation of any of the experiments and as part of the FRaT task protocol, rats were habituated to both the circular and triangular alternate contexts for a period of 8 min, respectively. The habituations involved free roaming with no introduction of any aversive stimuli. Habituations took place 72 h before the training phase of the FRaT task to ensure the rats are not able to retrospectively link the training session to the habituations (Zaki et al. 2023). All habituation sessions were recorded, and freezing was analyzed using ezTrack to ensure that there was no significant display or differences of freezing across contexts during the preexposures.

### Experiment 1

The purpose of this experiment was to determine whether a contextual fear memory can be updated with exposure to an alternate context following its reactivation. A group of 30 rats underwent a training session in the standard fear conditioning chamber before being divided into one of three groups based on the context in which they would be tested. The contexts were as follows (*n* = 10 each): same alternate context, different alternate context, or original fear chamber.

### Experiment 2

The purpose of this experiment was to determine whether the contextual fear memory updating that occurs in the FRaT task is reactivation dependent. For this experiment, 16 rats were used, and they were all tested in the same alternate context as the one they were exposed to post-reactivation. However, eight of the 16 rats were not placed back in the fear chamber on Day 2; that is, half of the rats were exposed to an alternate context but did not first experience reactivation of the original memory.

### Experiment 3

The purpose of this experiment was to determine whether the updating contextual fear memory is persistent. For this experiment, the same cohort from Experiment 2 was retested 4 weeks later in the same alternate context.

### Experiment 4

The purpose of this experiment was to determine whether the contextual fear memory updating occurs within a restricted time period following reactivation. There is evidence that the reconsolidation window for fear memory updating lasts for <6 h post-reactivation (Nader et al. 2000). For this experiment, the same FRaT protocol was used; however, groups of rats saw the alternate contexts either immediately after reactivation, 20 min after reactivation, or 6 h after reactivation (*n* = 8 per group). All rats were tested in the same alternate context they experienced on the reactivation day.

### Experiment 5

The purpose of this experiment was to determine the necessity of M1 mAChRs by targeting them with intracranial infusions of the selective M1 mAChR antagonist pirenzepine into the CA1 region of the dHPC. Thirty-two rats were obtained for this experiment. Rats were divided into one of four groups (Same-VEH, Different-VEH, Same-PIR, Different-PIR; Same = tested in same alternate context, different = tested in different alternate context). Each rat received a 2 min infusion of the drug or vehicle 30 min before the reactivation session.

### Data analysis

Freezing behavior in all contexts was analyzed using ezTrack, an open-source pipeline for scoring freezing behavior (Pennington et al. 2019). Freezing was defined as no movement except that required for breathing (Maren 2001). Freezing was additionally manually verified by the researcher for discrepancies across contexts. Video files were analyzed using ezTrack's analysis function, which bases each analysis on the session's duration and parameters. Freezing behavior was denoted as percent freezing out of 100% based on the timing of each session. Freezing behavior in alternate contexts was recorded using a video camera situated above each of the alternate contexts. One-way analysis of variance (ANOVA) was used to determine main group effects for Experiments 1–4. For Experiment 5, a 2 × 2 ANOVA was used. Tukey post hoc tests were conducted for multiple comparisons. In addition, independent samples *t*-tests were used for experiments with two groups. All statistical analyses were conducted with IBM SPSS (Version 29).
